# Supplementary material for: Adverse Pregnancy Outcomes after Multi-Professional Follow-Up of Women with Systemic Lupus Erythematosus: An Observational Study from a Single Centre in Sweden
Source: J Clin Med. 2020 Aug 11;9(8):2598. doi: 10.3390/jcm9082598 (PMC7464390; doi:10.3390/jcm9082598)
Supplement: Supplementary file 1 [file jcm-09-02598-s001.pdf]

Supplementary table 1 (Table S1). Associations between all investigated risk factors and the most frequent APO among all included pregnancies (59 conceptions, whereof 44 led to delivery). P-values, odds ratios and 95% confidence intervals are given.

**Risk factor**		**Total APO (*n*=33)**	**Pre-eclampsia and HELLP (*n*=12)**	**Miscarriage (<10 weeks) (*n*=13)**	**Preterm delivery (*n*=8)**	**Restricted foetal growth* (*n*=13)**
	Denominators	*n*=59	*n*=44	*n*=59	*n*=44	*n*=44
**Anti-dsDNA**	*Ever*	0.54 (OR 1.5, CI 0.4-5.5)	0.61 (OR 1.6, CI 0.3-8.4)	0.64 (OR 0.8, CI 0.2-2.5)	0.64 (OR 0.6, CI 0.1-4.2)	0.46 (OR 1.9, CI 0.4-9.6)
	≤1-year before	0.15 (OR 0.3, CI 0.1-1.5)	0.65 (OR 0.6, CI 0.1-4.3)	0.24 (OR 0.3, CI 0.04-2.2)	N.E.	0.53 (OR 0.6, CI 0.1-3.6)
	During pregnancy	0.86 (OR 0.9, CI 0.3-3.2)	0.45 (OR 1.8, CI 0.4-8.7)	N.T.	N.E.	0.89 (OR 0.9, CI 0.3-3.3)
**Anti-Ro/SSA**	*Ever*	0.24 (OR 0.5, CI 0.2-1.6)	0.14 (OR 0.2, CI 0.02-1.7)	0.36 (OR 0.5, CI 0.1-2.1)	0.35 (OR 0.3, CI 0.03-3.5)	0.75 (OR 0.7, CI 0.1-5.0)
	≤1-year before	0.14 (OR 0.4, CI 0.1-1.4)	N.E.	0.28 (OR 0.3, CI 0.04-2.5)	N.E.	0.58 (OR 0.5, CI 0.1-5.2)
	During pregnancy	0.39 (OR 0.6, CI 0.2-2.1)	0.16 (OR 0.2, CI 0.02-1.9)	N.T.	0.42 (OR 0.4, CI 0.04-4.0)	0.83 (OR 0.8, CI 0.1-5.5)
**Anti-La/SSB**	*Ever*	0.40 (OR 0.6, CI 0.2-1.9)	0.17 (OR 0.2, CI 0.02-2.0)	0.45 (OR 0.6, CI 0.1-2.4)	0.42 (OR 0.4, CI 0.03-4.0)	0.88 (OR 0.9, CI 0.1-6.0)
	≤1-year before	0.38 (OR 0.5, CI 0.1-2.2)	N.E.	0.41 (OR 0.4, CI 0.1-3.3)	N.E.	0.80 (OR 0.7, CI 0.1-7.9)
	During pregnancy	0.57 (OR 0.7, CI 0.2-2.5)	0.20 (OR 0.2, CI 0.02-2.2)	N.T.	0.49 (OR 0.4, CI 0.04-4.7)	0.97 (OR 1.0, CI 0.1-6.7)
**aCL**	*Ever*	0.40 (OR 1.7, CI 0.5-5.7)	0.51 (OR 1.8, CI 0.3-9.5)	0.08 (OR 3.9, CI 0.9-17.2)	0.31 (OR 2.7, CI 0.4-18.3)	0.83 (OR 0.8, CI 0.2-3.9)
	≤1-year before	0.74 (OR 0.8, CI 0.3-2.6)	0.33 (OR 0.3, CI 0.03-3.1)	0.47 (OR 1.4, CI 0.6-3.7)	0.12 (OR 4.8, CI 0.7-35.0)	0.61 (OR 1.5, CI 0.3-7.1)
	During pregnancy	0.46 (OR 1.6, CI 0.4-6.1)	N.E.	N.T.	**0.03 (OR=6.8, CI 1.2–39.4)**	0.19 (OR 2.9, CI 0.6-14.1)
**Anti-β2-GPI**	*Ever*	0.40 (OR 1.8, CI 0.5-7.4)	0.86 (OR 0.9, CI 0.2-4.8)	0.78 (OR 0.9, CI 0.3-2.6)	0.88 (OR 0.9, CI 0.1-5.6)	0.36 (OR 2.1, CI 0.4-10.9)
	≤1-year before	0.13	N.E.	0.66	0.78	0.61

		(OR 0.5, CI 0.2-1.3)		(OR 1.3, CI 0.4-4.1)	(OR 1.5, CI 0.1-19.9)	(OR 0.5, CI 0.05-5.9)
	During pregnancy	0.65 (OR 1.4, CI 0.4-5.4)	N.E.	N.T.	0.34 (OR 2.5, CI 0.4-15.7)	0.31 (OR 2.3, CI 0.5-10.7)
**LA test**	*Ever*	0.68 (OR 1.3, CI 0.4-4.3)	0.68 (OR 0.6, CI 0.1-6.5)	0.53 (OR 1.5, CI 0.4-5.0)	0.73 (OR 1.4, CI 0.2-12.0)	0.81 (OR 1.2, CI 0.2-6.4)
	≤1-year before	0.53 (OR 0.5, CI 0.1-4.3)	0.60 (OR 0.5, CI 0.05-5.4)	N.E.	0.70 (OR 1.8, CI 0.1-38.1)	0.49 (OR 0.4, CI 0.03-5.8)
	During pregnancy	0.36 (OR 2.7, CI 0.3-21.0)	0.43 (OR 2.7, CI 0.2-30.1)	N.T.	0.42 (OR 3.5, CI 0.2-72.6)	0.11 (OR 4.3, CI 0.7-26.7)
**Low C3**	*Ever*	0.88 (OR 0.9, CI 0.2-4.1)	0.38 (OR 0.5, CI 0.1-2.5)	0.19 (OR 0.5, CI 0.2-1.5)	0.27 (OR 0.3, CI 0.05-2.3)	0.93 (OR 0.9, CI 0.2-5.7)
	≤1-year before	0.37 (OR 0.6, CI 0.2-1.8)	0.95 (OR 1.0, CI 0.2-4.5)	0.10 (OR 0.3, CI 0.1-1.3)	0.86 (OR 1.2, CI 0.2-8.5)	0.57 (OR 1.6, CI 0.3-7.4)
	During pregnancy	0.66 (OR 0.8, CI 0.2-2.5)	0.81 (OR 1.2, CI 0.2-6.1)	N.T.	0.96 (OR 1.1, CI 0.2-6.4)	0.99 (OR 1.0, CI 0.2-4.2)
**Low C4**	*Ever*	0.91 (OR 0.9, CI 0.2-3.6)	0.96 (OR 1.1, CI 0.2-5.7)	0.35 (OR 0.6, CI 0.2-1.9)	0.86 (OR 0.8, CI 0.1-6.2)	0.81 (OR 1.2, CI 0.2-6.8)
	≤1-year before	0.48 (OR 1.5, CI 0.5-4.6)	0.48 (OR 1.8, CI 0.4-9.3)	0.69 (OR 0.8, CI 0.2-2.8)	0.77 (OR 1.3, CI 0.2-9.5)	0.48 (OR 1.8, CI 0.4-9.1)
	During pregnancy	0.69 (OR 1.3, CI 0.4-3.9)	0.96 (OR 1.0, CI 0.2-5.3)	N.T.	0.63 (OR 0.6, CI 0.1-4.1)	0.76 (OR 0.8, CI 0.2-3.5)
**Low C1q**	*Ever*	0.34 (OR 0.5, CI 0.2-1.9)	0.70 (OR 1.6, CI 0.2-16.2)	0.14 (OR 0.4, CI 0.1-1.3)	0.32 (OR 0.4, CI 0.04-2.8)	0.26 (OR 0.4, CI 0.1-2.1)
	≤1-year before	0.78 (OR 0.9, CI 0.3-2.4)	0.43 (OR 0.5, CI 0.1-2.7)	0.22 (OR 0.3, CI 0.05-2.0)	0.70 (OR 1.4, CI 0.2-9.4)	0.42 (OR 1.9, CI 0.4-8.7)
	During pregnancy	0.33 (OR 3.3, CI 1.0-11.11)	0.81 (OR 3.3, CI 1.0-11.11)	N.T.	0.96 (OR 3.3, CI 1.0-11.11)	0.41 (OR 1.9, CI 0.4-8.3)
**≥1 pos. aCL, anti-β2-GPI or LA test**	*Ever*	**0.05 (OR 3.3, CI 1.0–11.11)**	0.17 (OR 3.4, CI 0.6-19.6)	0.20 (OR 2.7, CI 0.6-11.8)	0.59 (OR 1.7, CI 0.2-11.8)	0.36 (OR 2.1, CI 0.4-10.2)
	≤1-year before	0.73 (OR 0.8, CI 0.3-2.6)	0.57 (OR 0.6, CI 0.1-3.3)	0.87 (OR 1.1, CI 0.4-3.1)	0.23 (OR 3.4, CI 0.5-24.5)	0.97 (OR 1.0, CI 0.2-5.3)
	During pregnancy	0.25 (OR 1.9, CI 0.7-5.5)	0.73 (OR 0.7, CI 0.1-6.4)	N.T.	0.72 (OR 1.4, CI 0.2-8.8)	0.26 (OR 2.1, CI 0.6-7.5)

**APS before pregnancy**	0.12 (OR 3.1, CI 0.8-12.7)	0.28 (OR 2.6, CI 0.5-13.9)	**0.04 (OR=3.3, CI 1.1–10.2)**	0.22 (OR 3.5, CI 0.5-26.2)	0.13 (OR 3.6, CI 0.7-18.5)
**Previous LN**	**0.005 (OR=5.9, CI 1.7–20.8)**	0.06 (OR 5.7, CI 0.9-34.6)	0.20 (OR 2.6, CI 0.6-10.7)	0.10 (OR 7.0, CI 0.7-71.5)	**0.01 (OR=16.6, CI 1.8–156.5)**
**Tobacco smoking until conception**	0.92 (OR 1.1, CI 0.2-6.0)	0.67 (OR 1.6, CI 0.2-12.3)	0.33 (OR 0.4, CI 0.05-2.8)	0.52 (OR 0.4, CI 0.04-5.0)	0.61 (OR 0.6, CI 0.1-3.7)

*IUGR, (intra-uterine growth restriction) and/or; SGA (small-for-gestational-age) new-borns and/or; LBW, (low birth weight). Yellow background indicates statistical significance. aCL, anti-cardiolipin antibody; anti-β2-GPI , anti-β2-glycoprotein I antibody; APS, antiphospholipid syndrome; CI, 95% confidence intervals; LA, lupus anticoagulant test; LN, lupus nephritis; N.E.; not estimated (used for calculations with division by zero); N.T., not tested; OR, odds ratio.

Supplementary table 2 (Table S2). Associations between all investigated risk factors and the most frequent APO among 1st pregnancies (26 conceptions, whereof 22 led to delivery). P-values, odds ratios and 95% confidence intervals are given.

**Risk factor**		**Total APO (*n*=15)**	**Pre-eclampsia and HELLP (*n*=7)**	**Miscarriage (<10 weeks) (*n*=4)**	**Preterm delivery (*n*=4)**	**Restricted foetal growth* (*n*=8)**
	Denominators	*n*=26	*n*=22	*n*=26	*n*=22	*n*=22
**Anti-dsDNA**	*Ever*	0.53 (OR 1.7, CI 0.3-5.3)	0.43 (OR 2.2, CI 0.3-15.0)	0.55 (OR 2.1, CI 0.2-23.3)	0.15 (OR 0.2, CI 0.01-2.0)	0.81 (OR 1.3, CI 0.2-7.4)
	≤1-year before	0.17 (OR 0.3, CI 0.04-1.8)	0.83 (OR 1.3, CI 0.2-9.8)	N.E.	N.E.	0.92 (OR 0.9, CI 0.1-6.8)
	During pregnancy	0.87 (OR 1.2, CI 0.2-7.1)	0.07 (OR 7.3, CI 0.9-61.3)	N.T.	N.E.	0.59 (OR 1.7, CI 0.3-11.3)
**Anti-Ro/SSA**	*Ever*	0.60 (OR 0.6, CI 0.2-3.4)	0.25 (OR 0.3, CI 0.03-2.6)	0.79 (OR 0.7, CI 0.1-8.2)	0.75 (OR 0.7, CI 0.1-7.9)	0.61 (OR 0.6, CI 0.1-4.2)
	≤1-year before	0.39 (OR 0.5, CI 0.1-2.7)	N.E.	0.80 (OR 0.7, CI 0.1-8.5)	N.E.	0.36 (OR 0.3, CI 0.03-3.6)
	During pregnancy	0.75 (OR 0.8, CI 0.1-4.3)	0.28 (OR 0.3, CI 0.03-2.9)	N.T.	0.95 (OR 0.9, CI 0.1-12.3)	0.66 (OR 0.6, CI 0.1-4.7)
**Anti-La/SSB**	*Ever*	0.97 (OR 1.0, CI 0.2-5.6)	0.36 (OR 0.3, CI 0.03-3.6)	0.93 (OR 0.9, CI 0.08-10.3)	0.91 (OR 0.9, CI 0.07-10.4)	0.86 (OR 0.8, CI 0.1-6.0)
	≤1-year before	0.86 (OR 1.2, CI 0.2-9.0)	N.E.	0.86 (OR 1.3, CI 0.1-15.5)	N.E.	0.75 (OR 0.7, CI 0.05-8.2)
	During pregnancy	0.87 (OR 1.2, CI 0.2-7.1)	0.41 (OR 0.4, CI 0.03-4.0)	N.T.	0.89 (OR 1.2, CI 0.1-16.4)	0.92 (OR 0.9, CI 0.1-6.8)
**aCL**	*Ever*	0.95 (OR 1.0, CI 0.2-4.5)	0.87 (OR 1.2, CI 0.2-7.1)	0.87 (OR 0.8, CI 0.1-7.0)	0.38 (OR 3.0, CI 0.3-34.6)	0.75 (OR 0.8, CI 0.1-4.3)
	≤1-year before	0.41	N.E.	N.E.	N.E.	0.64

		(OR 0.4, CI 0.1-3.2)				(OR 1.7, CI 0.2-14.1)
	During pregnancy	0.65 (OR 1.8, CI 0.1-23.4)	N.E.	N.T.	**0.03 (OR=32.0, CI 1.4–737.5)**	0.24 (OR 4.8, CI 0.4-65.8)
**Anti-β2-GPI**	*Ever*	0.24 (OR 3.0, CI 0.5-19.0)	0.67 (OR 1.5, CI 0.2-9.5)	N.E.	0.54 (OR 2.0, CI 0.2-17.9)	0.32 (OR 2.5, CI 0.4-15.2)
	≤1-year before	0.35 (OR 0.3, CI 0.02-3.8)	N.E.	N.E.	0.23 (OR 7.0, CI 0.3-162.2)	1.00 (OR 1.0, CI 0.1-13.9)
	During pregnancy	0.38 (OR 3.0, CI 0.3-34.6)	N.E.	N.T.	0.06 (OR 15.0, CI 0.9-251.1)	0.09 (OR 9.0, CI 0.7-113.0)
**LA test**	*Ever*	0.41 (OR 2.3, CI 0.3-15.8)	0.77 (OR 0.7, CI 0.1-8.1)	0.76 (OR 1.5, CI 0.1-20.3)	0.11 (OR 9.3, CI 0.6-139.6)	0.11 (OR 6.0, CI 0.7-53.7)
	≤1-year before	0.50 (OR 0.3, CI 0.01-8.2)	N.E.	N.E.	N.E.	N.E.
	During pregnancy	N.E.	0.44 (OR 3.3, CI 0.2-70.9)	N.T.	N.E.	N.E.
**Low C3**	*Ever*	0.08 (OR 5.4, CI 0.8-36.3)	0.36 (OR 3.0, CI 0.3-32.2)	0.93 (OR 1.1, CI 0.1-13.0)	0.91 (OR 1.2, CI 0.1-13.9)	0.26 (OR 3.9, CI 0.4-41.3)
	≤1-year before	0.77 (OR 1.3, CI 0.2-6.8)	0.34 (OR 2.7, CI 0.4-19.7)	N.E.	N.E.	**0.04 (OR=13.0, CI 1.2–152.2)**
	During pregnancy	1.00 (OR 1.0, CI 0.2-5.4)	0.50 (OR 2.0, CI 0.3-14.7)	N.T.	N.E.	0.29 (OR 0.4, CI 0.04-5.0)
**Low C4**	*Ever*	0.32	0.61	0.66	0.61	0.41

		(OR 2.3, CI 0.4-11.9)	(OR 1.7, CI 0.2-11.6)	(OR 1.7, CI 0.2-19.4)	(OR 1.9, CI 0.2-22.2)	(OR 2.3, CI 0.3-15.3)
	≤1-year before	0.06 (OR 6.4, CI 0.9-43.2)	**0.04 (OR=12.5, CI 1.1–143.4)**	0.71 (OR 0.6, CI 0.05-7.9)	N.E.	**0.02 (OR=20.0, CI 1.7–238.6)**
	During pregnancy	0.35 (OR 2.3, CI 0.4-13.6)	0.55 (OR 1.8, CI 0.3-12.5)	N.T.	0.33 (OR 3.7, CI 0.3-49.3)	0.26 (OR 3.0, CI 0.4-20.2)
**Low C1q**	*Ever*	0.29 (OR 0.4, CI 0.05-2.4)	N.E.	0.05 (OR 0.8, CI 0.01-1.0)	0.54 (OR 0.4, CI 0.03-6.4)	0.49 (OR 0.5, CI 0.05-4.2)
	≤1-year before	N.E.	0.69 (OR 0.7, CI 0.09-4.9)	N.E.	0.33 (OR 3.7, CI 0.3-49.3)	0.26 (OR 3.0, CI 0.5-20.2)
	During pregnancy	0.75 (OR 1.3, CI 0.2-7.6)	0.41 (OR 2.8, CI 0.3-30.9)	N.T.	N.E.	N.E.
**≥1 pos. aCL, anti-β2-GPI or LA test**	*Ever*	0.53 (OR 1.7, CI 0.3-8.3)	0.61 (OR 1.7, CI 0.2-11.6)	0.61 (OR 0.6, CI 0.07-4.5)	0.61 (OR 1.9, CI 0.2-22.2)	0.41 (OR 2.3, CI 0.3-15.3)
	≤1-year before	0.41 (OR 0.4, CI 0.06-3.2)	N.E.	N.E.	N.E.	0.64 (OR 1.7, CI 0.2-14.1)
	During pregnancy	0.22 (OR 4.4, CI 0.4-47.5)	0.63 (OR 0.6, CI 0.05-6.3)	N.T.	0.10 (OR 10.0, CI 0.7-149.0)	**0.03 (OR=17.3, CI 1.4–216.6)**
**APS before pregnancy**		0.17 (OR 5.0, CI 0.5-50.8)	0.14 (OR 4.9, CI 0.6-40.3)	0.92 (OR 1.1, CI 0.1-13.4)	0.17 (OR 5.0, CI 0.5-50.8)	**0.04 (OR=13.0, CI 1.1–152.4)**
**Previous LN**		0.13 (OR 3.5, CI 0.7-17.9)	0.29 (OR 2.9, CI 0.4-19.7)	0.87 (OR 0.8, CI 0.1-7.0)	0.38 (OR 3.0, CI 0.3-34.6)	**0.04 (OR=12.6, CI 1.2–133.9)**

**Tobacco smoking until conception**	0.96 (OR 1.1, CI 0.1-8.0)	0.38 (OR 2.8, CI 0.3-26.6)	0.64 (OR 1.9, CI 0.1-26.3)	N.E.	0.59 (OR 0.5, CI 0.04-6.0)

*IUGR, (intra-uterine growth restriction) and/or; SGA (small-for-gestational-age) new-borns and/or; LBW, (low birth weight). Yellow background indicates statistical significance. aCL, anti-cardiolipin antibody; anti-β2-GPI , anti-β2-glycoprotein I antibody; APS, antiphospholipid syndrome; CI, 95% confidence intervals; LA, lupus anticoagulant test; LN, lupus nephritis; N.E., not estimated (used for calculations with division by zero); N.T., not tested; OR, odds ratio.

Supplementary table 3 (Table S3). Associations between all investigated risk factors and the most frequent APO among subsequent pregnancies, not defined as 1st pregnancy after SLE diagnosis (33 conceptions, whereof 22 led to delivery). P-values, odds ratios and 95% confidence intervals are given.

**Risk factor**		**Total APO (n=18)**	**Pre-eclampsia and HELLP (n=5)**	**Miscarriage (<10 weeks) (n=9)**	**Preterm delivery (n=4)**	**Restricted foetal growth* (n=5)**
	Denominators	n=33	n=24	n=33	n=24	n=24
**Anti-dsDNA**	*Ever*	0.71 (OR 1.4, CI 0.3-7.5)	0.97 (OR 1.1, CI 0.1-10.3)	0.31 (OR 0.5, CI 0.1-2.0)	0.52 (OR 2.4, CI 0.2-34.1)	0.33 (OR 3.6, CI 0.3-44.6)
	≤1-year before	0.26 (OR 0.4, CI 0.08-2.0)	NE	0.70 (OR 0.6, CI 0.06-6.3)	N.E.	NE
	During pregnancy	0.61 (OR 0.7, CI 0.2-3.2)	0.42 (OR 0.4, CI 0.03-4.3)	N.T.	N.E.	0.42 (OR 0.4, CI 0.03-4.3)
**Anti-Ro/SSA**	*Ever*	0.24 (OR 0.3, CI 0.06-2.0)	N.E.	0.47 (OR 0.5, CI 0.06-3.5)	N.E.	0.88 (OR 0.8, CI 0.06-11.6)
	≤1-year before	0.19 (OR 0.2, CI 0.02-2.2)	N.E.	N.E.	N.E.	0.88 (OR 0.8, CI 0.06-11.6)
	During pregnancy	0.23 (OR 0.4, CI 0.04-5.0)	N.E.	N.T.	N.E.	0.88 (OR 0.8, CI 0.06-11.6)
**Anti-La/SSB**	*Ever*	0.24 (OR 0.3, CI 0.06-2.0)	N.E.	0.47 (OR 0.5, CI 0.06-3.5)	N.E.	0.88 (OR 0.8, CI 0.06-11.6)
	≤1-year before	0.19 (OR 0.2, CI 0.02-2.2)	N.E.	N.E.	N.E.	0.88 (OR 0.8, CI 0.06-11.6)
	During pregnancy	0.23 (OR 0.2, CI 0.02-2.6)	N.E.	N.T.	N.E.	0.88 (OR 0.8, CI 0.06-11.6)
**aCL**	*Ever*	0.17 (OR 3.3, CI 0.6-18.2)	0.33 (OR 3.6, CI 0.3-44.7)	N.E.	0.52 (OR 2.4, CI 0.2-34.1)	0.97 (OR 1.1, CI 0.1-10.3)
	≤1-year before	0.70 (OR 1.3, CI 0.3-5.9)	0.91 (OR 1.2, CI 0.08-16.2)	**0.01 (OR=3.8, CI 1.4–10.3)**	0.72 (OR 1.7, CI 0.1-26.2)	0.91 (OR 1.2, CI 0.08-16.2)
	During pregnancy	0.71 (OR 1.2, CI 0.5-2.6)	N.E.	N.T.	N.E.	N.E.
**Anti-β2-GPI**	*Ever*	0.85 (OR 1.1, CI 0.3-4.5)	N.E.	0.20 (OR 2.5, CI 0.6-10.2)	N.E.	0.91 (OR 1.2, CI 0.08-16.2)
	≤1-year before	0.41 (OR 0.6, CI 0.2-2.0)	N.E.	0.09 (OR 2.7, CI 0.9-8.2)	N.E.	N.E.

	During pregnancy	0.70 (OR 0.7, CI 0.1-3.7)	N.E.	N.T.	N.E.	N.E.
**LA test**	*Ever*	0.89 (OR 0.9, CI 0.3-3.3)	0.65 (OR 0.6, CI 0.04-7.4)	0.66 (OR 1.4, CI 0.3-5.5)	N.E.	N.E.
	≤1-year before	0.80 (OR 0.7, CI 0.03-14.2)	0.61 (OR 2.5, CI 0.07-87.0)	N.E.	N.E.	N.E.
	During pregnancy	0.83 (OR 0.8, CI 0.06-10.0)	0.60 (OR 2.2, CI 0.1-38.3)	N.T.	N.E.	N.E.
**Low C3**	*Ever*	0.12 (OR 0.3, CI 0.04-1.5)	**0.03 (OR=0.05, CI 0.004–0.7)**	0.11 (OR 0.3, CI 0.09-1.3)	0.09 (OR 0.1, CI 0.01-1.4)	0.18 (OR 0.2, CI 0.02-2.1)
	≤1-year before	0.08 (OR 0.3, CI 0.09-1.1)	0.28 (OR 0.3, CI 0.02-3.0)	0.36 (OR 0.5, CI 0.1-2.1)	N.E.	N.E.
	During pregnancy	0.27 (OR 0.5, CI 0.1-1.9)	0.55 (OR 0.5, CI 0.04-5.8)	N.T.	N.E.	N.E.
**Low C4**	*Ever*	0.40 (OR 0.5, CI 0.07-2.9)	0.70 (OR 0.6, CI 0.06-6.5)	0.13 (OR 0.3, CI 0.08-1.4)	0.49 (OR 0.4, CI 0.03-5.6)	0.70 (OR 0.6, CI 0.06-6.5)
	≤1-year before	0.45 (OR 0.6, CI 0.1-2.4)	0.29 (OR 0.3, CI 0.02-3.2)	0.75 (OR 0.8, CI 0.2-2.7)	N.E.	N.E.
	During pregnancy	0.63 (OR 0.7, CI 0.2-2.8)	0.43 (OR 0.4, CI 0.03-4.5)	N.T.	N.E.	N.E.
**Low C1q**	*Ever*	0.60 (OR 0.7, CI 0.1-3.1)	0.65 (OR 0.5, CI 0.04-7.7)	0.69 (OR 1.3, CI 0.4-4.4)	0.36 (OR 0.3, CI 0.02-4.9)	0.16 (OR 0.1, CI 0.01-2.1)
	≤1-year before	0.15 (OR 0.3, CI 0.05-1.6)	N.E.	0.90 (OR 1.1, CI 0.2-6.0)	N.E.	N.E.
	During pregnancy	0.05 (OR 0.2, CI 0.4-1.0)	0.18 (OR 0.2, CI 0.01-2.2)	N.T.	0.33 (OR 0.3, CI 0.02-3.8)	0.18 (OR 0.2, CI 0.01-2.2)
**≥1 pos. aCL, anti-β2-GPI or LA test**	*Ever*	**0.04 (OR=11.3, CI 1.1–115.4)**	N.E.	N.E.	0.77 (OR 1.5, CI 0.1-22.0)	0.55 (OR 2.2, CI 0.2-28.2)
	≤1-year before	0.76 (OR 1.3, CI 0.3-5.3)	0.51 (OR 2.2, CI 0.2-22.1)	0.17 (OR 2.3, CI 0.7-7.1)	0.92 (OR 0.9, CI 0.06-12.5)	0.69 (OR 0.6, CI 0.05-7.6)
	During pregnancy	0.73 (OR 1.3, CI 0.4-4.5)	0.87 (OR 0.8, CI 0.06-10.7)	N.T.	N.E.	N.E.
**APS before pregnancy**		0.21	0.69	**0.02 (OR=4.9,**	0.48	0.69

	(OR 2.8, CI 0.6-13.4)	(OR 1.6, CI 0.2-15.8)	**CI 1.3–17.7)**	(OR 2.6, CI 0.2-36.1)	(OR 1.6, CI 0.2-15.8)
**Previous LN**	**0.01 (OR=12.0, CI 1.8–82.0)**	N.E.	**0.04 (OR=5.7, CI 1.1–31.1)**	N.E.	N.E.
**Tobacco smoking until conception**	0.90 (OR 1.1, CI 0.2-6.7)	0.89 (OR 0.8, CI 0.06-11.8)	N.E.	0.89 (OR 1.2, CI 0.08-19.5)	0.89 (OR 0.8, CI 0.06-11.8)

*IUGR, (intra-uterine growth restriction) and/or; SGA (small-for-gestational-age) new-borns and/or; LBW, (low birth weight). Yellow background indicates statistical significance. aCL, anti-cardiolipin antibody; anti-β2-GPI , anti-β2-glycoprotein I antibody; APS, antiphospholipid syndrome; CI, 95% confidence intervals; LA, lupus anticoagulant test; LN, lupus nephritis; N.E., not estimated (used for calculations with division by zero); N.T., not tested; OR, odds ratio.
